# Hepatic arterial infusion chemotherapy versus sorafenib for advanced hepatocellular carcinoma with portal vein tumor thrombus: An updated meta-analysis and systematic review

**DOI:** 10.3389/fonc.2023.1085166

**Published:** 2023-01-27

**Authors:** Wei Zhang, Deliang Ouyang, Zhangkan Huang, Xu Che

**Affiliations:** ^1^ Department of Hepatobiliary and Pancreatic Surgery, National Cancer Center/National Clinical Research Center for Cancer/Cancer Hospital & Shenzhen Hospital, Chinese Academy of Medical Sciences and Peking Union Medical College, Shenzhen, Guangdong, China; ^2^ Department of General Surgery, The Third Affiliated Hospital, Hengyang Medical School, University of South China, Hengyang, Hunan, China

**Keywords:** hepatocellular carcinoma, portal vein tumor thrombosis, sorafenib, hepatic arterial infusion chemotherapy, prognosis, meta-analysis

## Abstract

**Background:**

Sorafenib was the first drug approved for advanced hepatocellular carcinoma (HCC). However, it is limited by poor efficacy for HCC with portal vein tumor thrombus (PVTT). Some studies suggested that hepatic artery infusion chemotherapy (HAIC) could provide survival benefits to patients with advanced HCC with PVTT.

**Aim:**

The study aimed to compare the efficacy of HAIC versus sorafenib in patients with HCC accompanied by PVTT.

**Methods:**

The PubMed, Embase, and Cochrane Library databases were searched for studies published until September 2022. Statistical analyses were performed using Stata SE 15 software.

**Results:**

Eight studies with 672 patients, 403 in the HAIC group and 269 in the sorafenib group, were included in the meta-analysis. The rates of complete response (RR=3.88, 95%CI:1.35-11.16, P=0.01), partial response (RR=3.46, 95%CI:1.94-6.18, P<0.0001), objective response rate (RR=4.21, 95%CI:2.44-7.28, P<0.00001) and disease control rate (RR=1.73, 95%CI:1.28-2.35, P=0.0004) were significantly higher in the HAIC group compared to the sorafenib group, whereas the progressive disease rate (RR=0.57, 95%CI:0.40-0.80, P=0.02) was significantly lower in the former. In contrast, the stable disease rate (RR=1.10, 95%CI (0.69-1.76), P=0.68) was similar in both groups. The overall survival (HR=0.50, 95%CI:0.40-0.63, P<0.05) and progression-free survival (HR=0.49, 95%CI:0.35-0.67, P<0.05) rates were significantly higher in the HAIC group compared to the sorafenib group.

**Conclusion:**

HAIC has better efficacy against HCC with PVTT than sorafenib and may be considered an alternative to the latter. However, more high-quality randomized control trials and longer follow-ups are needed to verify our findings.

## 1 Introduction

Primary liver cancer is a highly aggressive malignancy, ranking sixth and fourth in incidence and mortality rates, respectively, worldwide (1). Its major cause is liver cirrhosis (LC), including alcoholic cirrhosis, viral-related cirrhosis, cryptogenic cirrhosis, and other types (2). Over 85%-90% of the primary liver cancer cases are hepatocellular carcinoma (HCC). LC and HCC are highly prevalent in Europe and are associated with high mortality rates. However, LC mortality is decreasing, while HCC mortality is increasing significantly (3). Most patients with HCC are diagnosed at advanced stages, which precludes the possibility of radical surgical resection (4, 5). Owing to the biological characteristics of HCC and the anatomical features of the liver, tumor cells are prone to invading the intrahepatic vascular system, particularly the portal vein system. Portal vein tumor thrombus (PVTT) is seen in 10-62.2% of HCC cases (6–8) and accelerates disease progression, with intra- and extra-hepatic metastases, portal hypertension, jaundice, and peritoneal effusion. Not surprisingly, the median survival duration of HCC patients with PVTT is only 2-12 months (9–11). PVTT is a major adverse prognostic factor for HCC and is recognized as a significant determinant in the clinical staging system (8, 12–14).

Currently, sorafenib is the first-line drug recommended by the American Association for the Study of Liver Diseases (AASLD), the Asian-Pacific Association for the Study of the Liver (APASL), and the European Association for the Study of the Liver (EASL) for the treatment of HCC with PVTT, despite its low efficacy (15–17). Owing to the adverse events associated with long-term sorafenib treatment, it is given at a lower dose or altogether discontinued in many patients, which limits its therapeutic potential. Once sorafenib is ineffective or discontinued, the formation of PVTT is markedly accelerated, decreasing the portal blood flow and leading to rapid liver function deterioration, eventually complicating the administration of second-line therapy. Therefore, a more effective treatment is needed for HCC patients with PVTT.

Hepatic artery infusion chemotherapy (HAIC) was developed in Japan and is used to treat patients with advanced HCC. HAIC increases intra-tumoral drug concentrations compared to systemic chemotherapy, resulting in greater therapeutic efficacy and fewer adverse effects. Several studies conducted in Asian countries have shown good HAIC efficacy in HCC patients with PVTT (18–21). Therefore, we conducted this meta-analysis to systematically assess whether HAIC could be an alternative treatment option for advanced HCC with PVTT.

## 2 Materials and methods

A systematic review and meta-analysis were conducted based on the PRISMA guidelines for preferred reporting items (22). This study was exempted from the requirement to obtain formal institutional review board approval or informed consent from patients since it was a secondary study with publicly available data. This meta-analysis was registered on PROSPERO (https://www.crd.york.ac.uk/PROSPERO/) with the registration number CRD42022367379.

### 2.1 Search strategy

The EMBASE, PubMed, and Cochrane Library databases were searched for articles published until September 2022. The search strategy is detailed in **Supplementary File 1**. The reference lists of the included studies were also manually screened for additional studies. The authors were contacted when necessary to obtain additional data. Only the highest quality studies were selected for multiple studies from the same author or medical center, and duplications in sample size.

### 2.2 Inclusion criteria

(i) Confirmed HCC with PVTT in the study population; (ii) comparing the efficacy of HAIC and sorafenib; (iii) conducted on human subjects; and (iv) reporting tumor response rates, adverse events, and long-term survival outcomes. There were no restrictions on the sample size, follow-up duration, or publication language.

### 2.3 Extraction criteria

(i) Incomplete information and unresponsive authors, lack of peer review; (ii) single-arm studies of HAIC or sorafenib; (iii) administration of other treatments such as combined TACE or radiofrequency ablation; and (iv) robotic studies, reviews, case reports, and animal studies.

### 2.4 Quality assessment

A risk assessment of randomized controlled trials (RCTs) was conducted according to the risk assessment tool recommended by the Cochrane Collaboration Network. The quality of the cohort studies was evaluated based on the Newcastle-Ottawa Scale (NOS). The quality assessment is attached to **Supplementary File 2**.

### 2.5 Statistical analysis

Stata SE 15 software was used for the statistical analyses. Relative risk (RR) was calculated to compare binary variables by the Mantel-Haenszel method. Weighted mean difference (WMD) was calculated to compare continuous variables by the inverse variance method. Heterogeneity among studies was qualitatively evaluated using the *χ2*-based Q test and *I* ^2^ statistics. *I2* < 30%, 30% ≤ *I2 ≤* 50%, and *I2* > 50% are indicative of low, moderate, and high heterogeneity, respectively. Random models were used in this meta-analysis. Sensitivity analysis was performed by removing one study at a time to assess the effect of the individual studies on the results. When the sensitivity analysis was performed, the results with low heterogeneity among studies were considered the results of this study. The studies that were not included in the statistical analysis are described in the Results section. Funnel plots were used to assess publication bias qualitatively, and quantitative assessment was performed using Begg’s and Egger’s tests (**Supplementary File 3**). P < 0.05 was considered statistically significant.

## 3 Results

### 3.1 Search results and study selection

**Figure 1:**
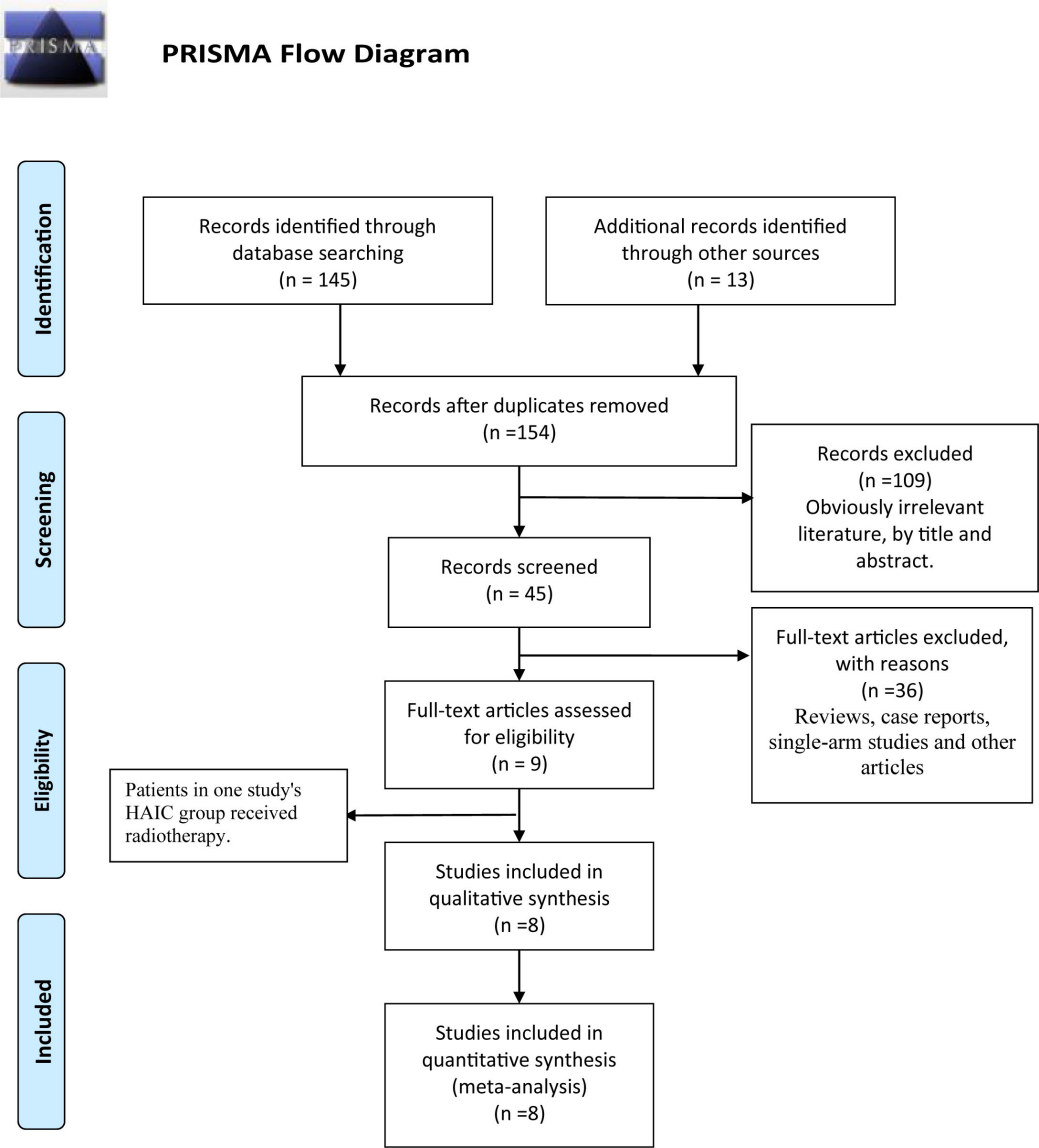
PRISMA flow diagram.

A total of 78 articles were retrieved, of which 35 remained after removing the duplicates. Following the exclusion of reviews, case reports, and other non-statistical literature, eight articles were finally included in the meta-analysis. A flowchart outlining the literature search is shown in **Figure 1**. The eight studies included 672 patients, of which 403 (59.97%) underwent HAIC, and 269 (40.03%) had received sorafenib. The characteristics of these studies are summarized in **Table 1**. The clinical features and outcomes of the two groups are presented in **Table 2**.

**Table 1:** Basic characteristics of the included studies.

Study	Country	Study period	Medical center	Study type	Case	Gender		Age		Quality
					HAIC vs. sorafenib	HAIC	Sorafenib	HAIC	Sorafenib	
Abdelmaksoud 2021 (21)	Egypt	2016-2017	Cairo University	CCS	20 vs. 29	18/2	26/3	NA	NA	5
Ahn 2021 (22)	Korea	2008-2016	Korea University Medical Center	R	38 vs. 35	30/8	30/5	53.0±11.6	58.3±9.5	6
Choi 2018 (23)	Korea	2013-2015	Six university hospitals	RCT	29 vs. 29	25/4	27/2	60.3±9.5	60.2±7.3	*
Moriguchi 2017 (24)	Japan	2002-2013	Shizuoka Cancer Center	R	32 vs. 14	12/2	29/3	68 (53–82)	65 (40–81)	7
Song 2015 (14)	Korea	2018-2013	Seven Korean tertiary medical centers	R	50 vs. 60	38/12	44/16	54.3±9.9	55.8±9.0	7
Yang 2017 (25)	Korea	2005-2013	Seoul St.Mary’s HospitalKorea	CCS	54 vs. 53	50/4	39/14	54.4±11.0	58.0±9.2	8
Nakano 2017 (26)	Japan	2008-2014	Kurume University School of Medicine	P	44 vs. 20	33/11	17/3	63.4±10.0	65.4±8.1	7
Kawaoka 2015 (27)	Japan	2000-2010	Division of Frontier Medical Science, Hiroshima University	R	136 vs. 41	123/13	29/12	67(30–85)	69(30–81)	6

R, Retrospective cohort studies; P, Prospective cohort studies; CCS, Case-control studies; RCT, randomized controlled trial; HAIC, Hepatic Artery Infusion Chemotherapy; NA, Not available.

**Table 2:** Clinical characteristics and clinical outcomes of the two groups included in the study.

Study	Country	HAIC's	PVTT type	PVTT case, n (%)		Focal lesions*		Platelet count, ×10^9^/L		AlT, IU/ml		AST, IU/mL	
		agents		HAIC	Sorafenib	HAIC	Sorafenib	HAIC	Sorafenib	HAIC	Sorafenib	HAIC	Sorafenib
Abdelmaksoud 2021	Egypt	C/Ad	Vp1/Vp2/Vp3/Vp4	NA	NA	13/20	14/29	NA	NA	50.73±31.16	91.31±141.04	53±24.88	116.72± 84.07
Ahn 2021	Korea	C/5-FU	Vp3/Vp4	NA	NA	14/24	16/19	162±83	196±105	89±63	103±98	89±63	103±98
Choi 2018	Korea	C/5-FU	Vp3/Vp4	10/19	11/18	13/16	10/19	NA	NA	NA	NA	NA	NA
Moriguchi 2017	Japan	C/5-FU	Vp3/Vp4	25/7	5/9	NA	NA	15.7(7.3–45.9)	12.4(10.6–24.3)	NA	NA	NA	NA
Song 2015	Korea	C/5-FU	Vp2/Vp3/Vp4	7/14/29	5/16/39	NA	NA	NA	NA	NA	NA	NA	NA
Yang 2017	Korea	C/5-FU	Vp2/Vp3/Vp4	3/33/18	12/17/24	8/46	17/36	NA	NA	NA	NA	NA	NA
Nakano 2017	Japan	C/5-FU	Vp3+Vp4/Vp1+Vp2	5/15	4/40	NA	NA	NA	NA	NA	NA	NA	NA
Kawaoka 2015	Japan	C/5-FU	Vp2/Vp3/Vp4	29/36/27	2/8/4	NA	NA	122(46–888)	153(53–207)	NA	NA	NA	NA
Study	Lesion size (cm)			Total bilirubin, mg/dL		Child-Pugh class, A/B		Previous treatment**		AFP, ng/mL		Cause of HCC***	
	HAIC		Sorafenib	HAIC	Sorafenib	HAIC	Sorafenib	HAIC	Sorafenib	HAIC	Sorafenib	HAIC	Sorafenib
Abdelmaksoud 2021	5.19±2.66		7.38±3.78	0.93±0.52	2.02±1.87	NA	NA	NA	NA	NA	NA	NA	NA
Ahn 2021	NA		NA	1.04±0.48	1.16±0.55	A/B:27/11	24/11	29/9	21/14	71341±14823	69,745±21,274	33/2/2/1	24/2/6/3
Choi 2018	<10cm/>10cm:14/15		12/17	NA	NA	A/B:27/2	25/4	9/20	3/26	260.0(3.6–84604.6)	130.8 (2.0–225971)	21/0/5/3	18/5/6/0
Moriguchi 2017	74.7 (0–179.1)		65.8(32.7–108.0)	0.7(0.3–1.4)	0.8(0. –1.4)			12/20	9/5	466.1(5.1–340,140)	416.9 (4.3–211,634)	12/7/13	4/8/2
Song 2015	<10cm/>10cm:22/28		31/29	NA	NA	A/B:45/5	47/13	18/32	9/51	<200/>200:15/35	20/38	44/2/3/1	41/5/8/6
Yang 2017	12.5±4.6		9.2±5.1	NA	NA	A/B:25/29	34/19	27/27	37/16	<400/>400:23/31	23/30	44/6/3/1	43/4/3/3
Nakano 2017	NA		NA	NA	NA	A	A	5/15	4/40	<1000:12/8	24/20	5/8/7	8/29/7
Kawaoka 2015	45 (10–180)		40 (10–190)	NA	NA	NA	NA	NA	NA	415.3(2.6–1 938 000)	208.0(3–85 632)	33/75/28	15/24/2

*Single/multiple; **None/curative (Resection, TACE, RFA), n; ***Etiology, HBV/HCV/alcohol/cryptogenic (or HBV/HCV/alcohol/others), HBV, hepatitis B virus; HCV, hepatitis C virus, n; HAIC, Hepatic Artery Infusion Chemotherapy; AlT, aspartate aminotransferase; AST, alanine aminotransferase; AFP, α-fetoprotein; BCLC, Barcelona Clinic Liver Cancer Staging classification; C/Ad, Cisplatin and Adriamycin; C/5-FU, Cisplatin and 5-fluorouracil; NA, Not available.

### 32 Results of the meta-analysis

Eight measurable outcomes were calculated to compare the efficacy of HAIC and sorafenib. All outcomes are summarized in **Table 3**.

**Table 3:** Meta-analysis results of all available studies in measured outcomes.

Measured Outcomes	No. Studies	No. Patients	Heterogeneity Test		Model	RR/HR	95% CI	*P*
		HAIC vs. Sorafenib	I^2^(%)	*P*				
Partial response	8	403 vs. 269	1	0.42	Random	3.46	1.94,6.18	<0.0001
Complete response	8	403 vs. 269	0	0.95	Random	3.88	1.35,11.16	0.01
Stable disease	8	403 vs. 269	77	<0.0001	Random	1.1	0.69,1.76	0.68
Progressive disease	8	403 vs. 269	68	0.002	Random	0.54	0.38,0.78	0.0009
Objective response rate	8	403 vs. 269	2	0.41	Random	4.21	2.44,7.28	<0.00001
Disease control rate	8	403 vs. 269	74	0.0003	Random	1.73	1.28,2.35	0.0004
Overall survival	7	383 vs. 240	0	0.705	Random	0.5	0.40,0.63	<0.05
Disease free survival	5	193 vs. 158	25.6	0.251	Random	0.49	0.35,0.67	<0.05

No., the number of; HAIC, Hepatic Artery Infusion Chemotherapy; RR/HR, relative risk/Hazard ratio; CI, Confidence interval; Bold indicates statistical significance.

#### 32.1 Tumor response rate

**Figure 2:**
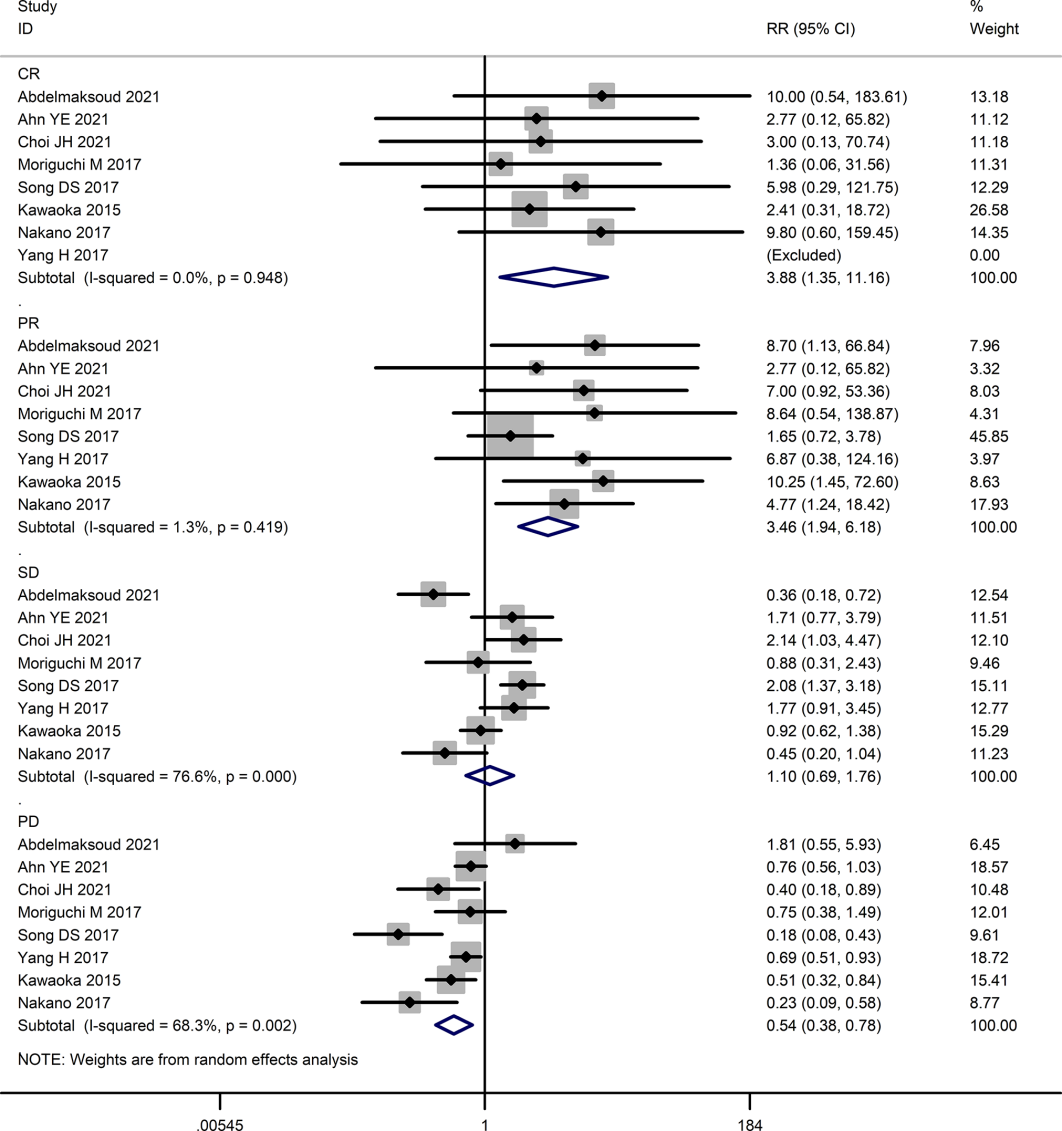
Forest map of complete response **(CR)**, partial response **(PR)**, stable disease **(SD)**, and progressive disease **(PD)**.

**Figure 3:**
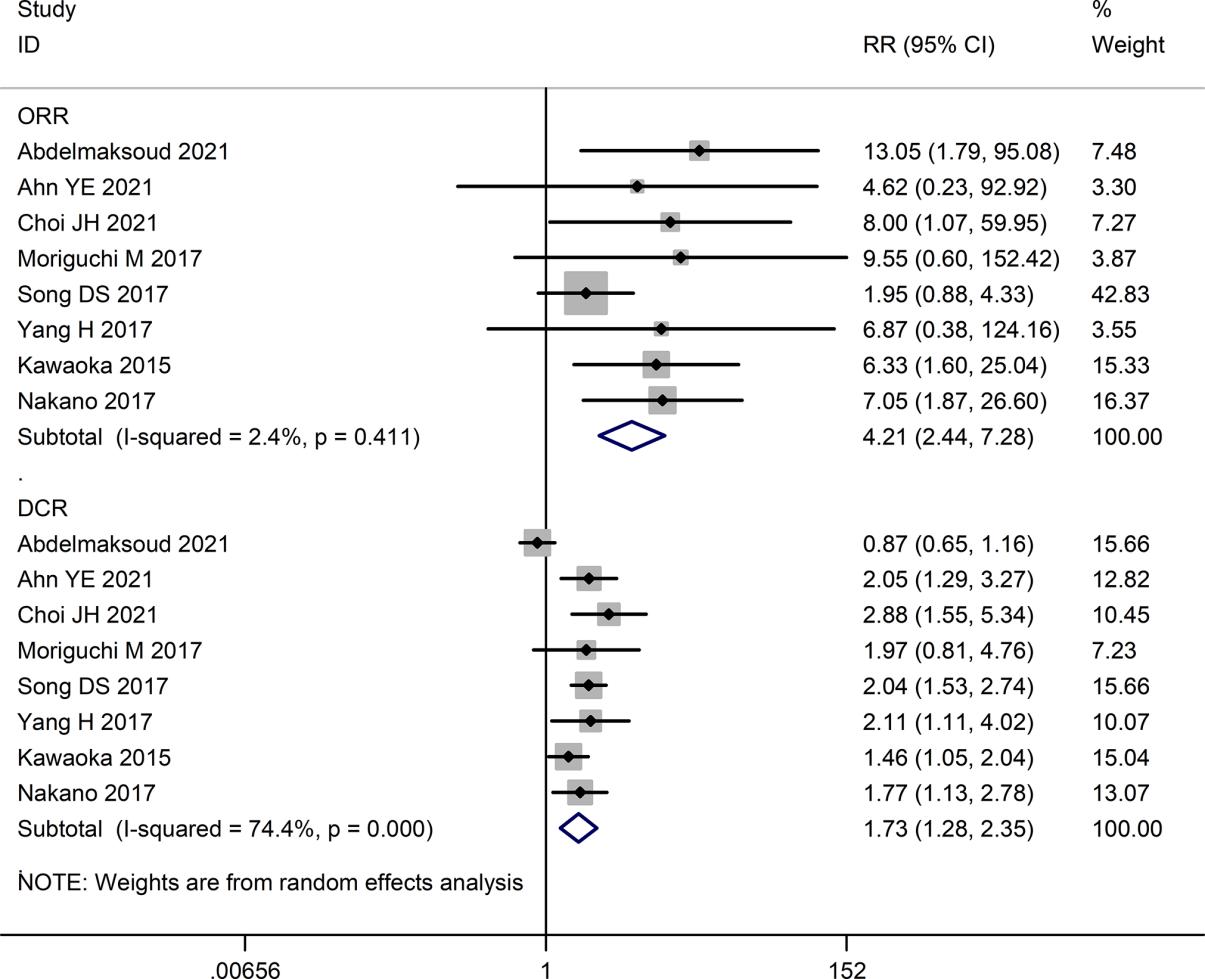
Forest map of objective response rate **(ORR)** and disease control rate **(DCR)**.

The tumor response rates of HAIC and sorafenib were compared in six studies (16, 23–27). The pooled effect sizes were calculated using appropriate models. The partial response (PR) rate (RR=3.46, 95%CI:1.94, 6.18, P<0.0001), complete response (CR) rate (RR=3.88, 95%CI:1.35, 11.16, P=0.01), stable disease (SD) rate (RR=1.31, 95% CI:0.74, 2.32, P=0.36), objective response rate (ORR) (RR=4.21, 95% CI:2.44, 7.28, P<0.00001), and disease control rate (DCR) (RR=1.73, 95% CI:1.28, 2.35, P=0.0004) were significantly higher in the HAIC group compared to the sorafenib group. Consistent with this, the progressive disease (PD) rate was lower in the HAIC group (RR=0.57, 95%CI:0.40, 0.80, P=0.02). In contrast, the stable disease (SD) rate showed no significant difference between the two groups (RR=1.10, 95%CI:0.69, 1.76, P=0.68). The Forest plots are depicted in **Figures 2**, **3**.

#### 3.2.2 Long-term outcomes

**Figure 4:**
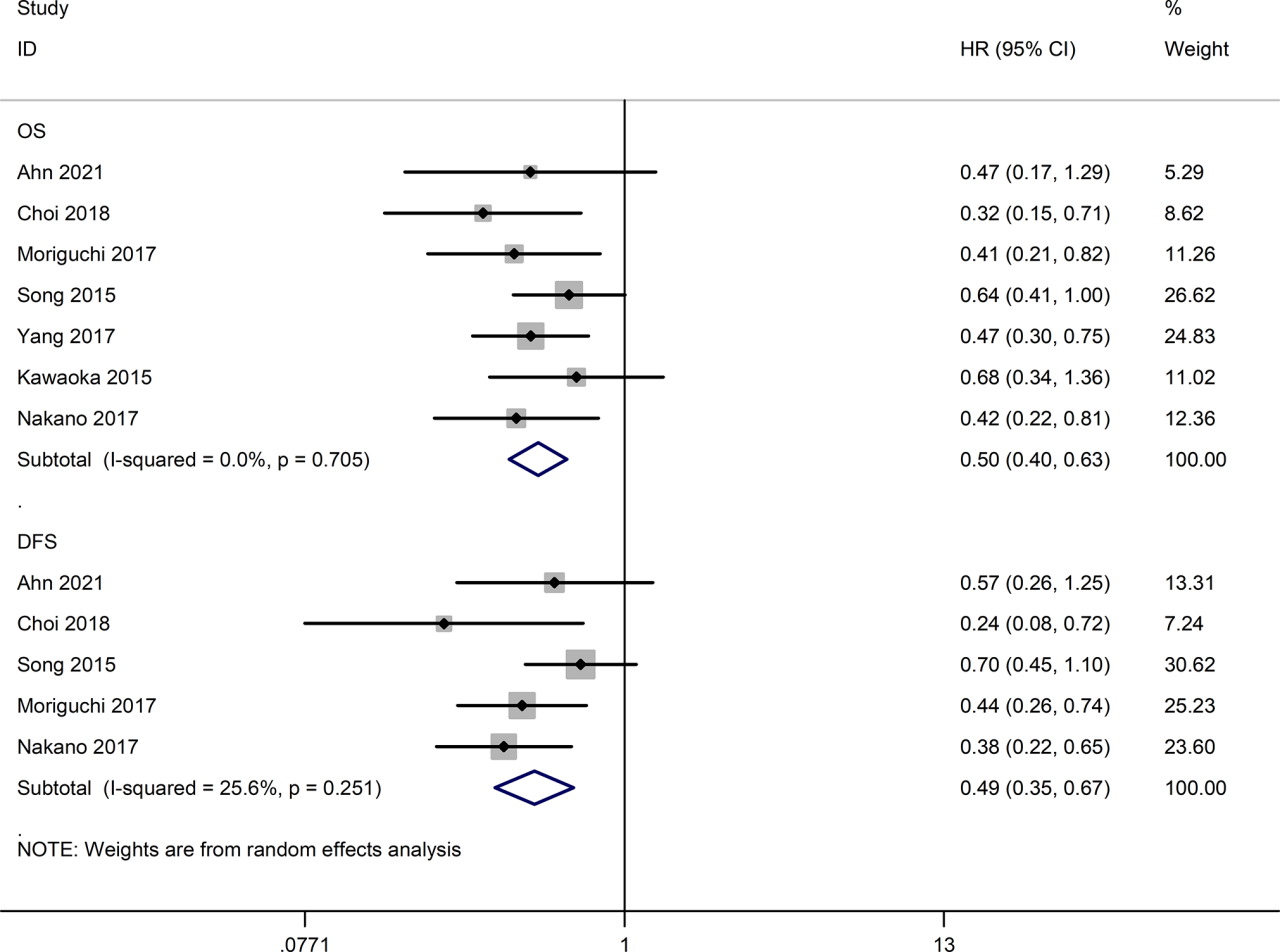
Forest map of overall survival and disease-free survival.

Seven studies (16, 24–29) reported long-term outcomes. As shown in **Figure 4**, the HAIC group had better overall survival (OS) (HR=0.50, 95% CI:0.40,0.63, P<0.05) and disease-free survival (DFS) (HR=0.49, 95% CI:0.35,0.67, P<0.05) compared to the sorafenib group.

#### 3.2.3 Adverse events

The adverse events of HAIC were associated with chemotherapy. These included leukopenia, neutropenia, thrombocytopenia, and jaundice/hyperbilirubinemia. In contrast, the adverse events of sorafenib were associated with extra-tumoral targeted sites, such as hand and foot syndrome, fatigue, diarrhea, etc. Therefore, a comparison of the adverse events of both treatments was not assessed. We summarized the adverse events of both groups in **Table 4**.

**Table 4:** Adverse Events Related to Treatment.

Adverse events	Abdelmaksoud 2021		Ahn 2021		Choi 2018		Moriguchi 2017		Nakano 2017		Song 2015		Yang 2017	
	HAIC	sorafenib	HAIC	sorafenib	HAIC	sorafenib	HAIC	sorafenib	HAIC	sorafenib	HAIC	sorafenib	HAIC	sorafenib
Cases in the study (n, %)	20	29	38	35	29	29	32	14	44	20	50	60	54	53
Abdominal pain	NA	0	2(5.2)	3(8.6)	NA	NA	NA	NA	NA	NA	NA	NA	NA	NA
Nausea	NA	0	4(10.5)	7(20)	NA	1(3.4)	2(6.3)	0	NA	NA	31(62)	NA	NA	NA
Hand and foot syndrome	NA	12(41.4)	0	10(28.6)	NA	9(31)	0	1(7.1)	NA	NA	NA	27(45)	NA	15(28.3)
Weight loss	NA	7(24.1)	NA	NA	NA	NA	NA	NA	NA	NA	NA	NA	NA	9(10.8)
Fatigue	NA	5(17.2)	3(7.9)	10(28.6)	NA	NA	0	3(21.4)	NA	NA	NA	22(36.7)	NA	NA
Elevated liver enzymes	NA	2(6.9)	6(15.8)	8(22.9)	10(34.5)	8(27.6)	1(3.1)	2(14.3)	NA	NA	36(72)	NA	9(16.7)	NA
Jaundice/Hyperbilirubinemia	NA	1(3.4)	6(15.8)	13(37.1)	13(44.8)	10(34.5)	NA	NA	NA	NA	37(74)	NA	16(29.6)	NA
Thrombocytopenia	NA	NA	17(44.7)	3(8.6)	NA	NA	8(25)	1(7.14)	NA	NA	42(84)	NA	12(22.2)	NA
Anemia	NA	NA	14(36.8)	7(20)	2(6.9)	NA	NA	NA	NA	NA	49(98)	NA	NA	NA
Diarrhea	NA	NA	2(5.3)	6(17.1)	2(6.9)	5(17.2)	0	1(7.14)	NA	NA	NA	23(38.3)	NA	NA
Neutrophil count decreased	NA	NA	9(23.7)	1(2.9)	NA	NA	4(12.5)	0	NA	NA	31(62)	NA	NA	NA
Leukopenia	NA	NA	NA	NA	NA	NA	NA	NA	NA	NA	NA	NA	26(48.1)	NA

HAIC, Hepatic Artery Infusion Chemotherapy; NA, Not available.

### 3.3 Subgroup analysis

We performed a subgroup analysis by countries, types of PVTT, and chemotherapeutic agents for HACI. We found that HAIC was more effective than sorafenib, independent of the study population (Korean, Japanese, or Egyptian) and the type of PVTT. However, patients with PVTT of the portal trunk and its first branches (Vp4 and Vp3) had better prognoses than patients with terminal portal veins (Vp2), with higher DCR rates, lower PR rates, and higher OS and DFS. The results are presented in **Table 5**.

**Table 5:** Subgroup analysis results of all available studies in measured outcomes.

Measured Outcomes	Subgroup	No. Studies	Heterogeneity Test		Model	RR/HR	95% CI	*P*
			*I^2^* (%)	*P*				
Objective response	Korea	4	0	0.482	Random	2.6	1.30,5.23	**<0.05**
	Japan	3	0	0.966	Random	6.95	2.82,17.15	**<0.05**
	Vp3/Vp4	3	0	0.934	Random	7.40	1.77,30.98	**<0.05**
	Vp2/Vp3/Vp4	3	31.6	0.232	Random	3.23	1.24,8.41	**<0.05**
	HAIC: C/5-FU	7	0	0.45	Random	3.76	2.16,6.53	**<0.00001**
Disease control	Korea	4	0	0.796	Random	2.14	1.72,2.66	**<0.05**
	Japan	3	0	0.706	Random	1.59	1.24,2.06	**<0.05**
	Vp3/Vp4	3	0	0.658	Random	2.26	1.61,3.19	**<0.05**
	Vp2/Vp3/Vp4	3	19.6	0.288	Random	1.80	1.41,2.29	**<0.05**
	HAIC: C/5-FU	7	0	0.59	Random	1.89	1.61,2.23	**<0.00001**
Progressive disease	Korea	4	79.5	0.002	Random	0.5	0.30,0.85	**<0.05**
	Japan	3	51.7	0.126	Random	0.48	0.27,0.85	**<0.05**
	Vp3/Vp4	3	19	0.291	Random	0.68	0.49,0.95	**<0.05**
	Vp2/Vp3/Vp4	3	81.6	0.004	Random	0.44	0.22,0.88	**<0.05**
	HAIC: C/5-FU	7	70	0.003	Random	0.5	0.35,0.72	**0.0002**
Overall survival	Korea	4	0	0.482	Random	0.51	0.38,0.68	**<0.05**
	Japan	3	0	0.521	Random	0.49	0.33,0.72	**<0.05**
	Vp3/Vp4	3	0	0.822	Random	0.39	0.25,0.62	**<0.05**
	Vp2/Vp3/Vp4	3	0	0.3561	Random	0.57	0.43,0.77	**<0.05**
	HAIC: C/5-FU	7	0	0.705	Random	0.5	0.40,0.63	**<0.05**
Disease free survival	Korea	3	38.1	0.199	Random	0.54	0.32,0.93	**<0.05**
	Japan	2	0	0.701	Random	0.41	0.28,0.60	**<0.05**
	Vp3/Vp4	3	0	0.441	Random	0.43	0.29,0.65	**<0.05**
	Vp2/Vp3/Vp4	1	–	–	Random	0.70	0.45,1.10	**>0.05**
	HAIC: C/5-FU	7	25.6	0.251	Random	0.49	0.35,0.67	**<0.05**

No., the number of; HAIC, Hepatic Artery Infusion Chemotherapy; RR/HR, relative risk/Hazard ratio; CI, Confidence interval; Bold indicates statistical significance. C/Ad- Cisplatin and Adriamycin; C/5-FU- Cisplatin and 5-fluorouracil.

### 3.4 Sensitivity analysis and publication bias

The sensitivity analysis showed that the results of each outcome were stable. Begg’s funnel plots with pseudo 95% confidence limits were symmetrical, which suggested limited or no publication bias *(* **Supplementary File 3**). We did not detect publication bias using Begg’s and Egger’s tests (**Supplemental File 4**).

## 4 Discussion

Patients with advanced HCC, especially those presenting PVTT, have a poor prognosis due to limited treatment options. PVTT can reduce intrahepatic blood flow and cause portal hypertension, leading to impaired liver function and fatal consequences. These involve increased ascites, esophagogastric fundic varices, and ruptured hemorrhage. PVTT is considered an indicator of poor prognosis in HCC, and various treatment options have been explored to improve overall survival in this setting (10, 30).

HAIC has evolved from transcatheter arterial infusion (TAI). Different combination chemotherapy agents are currently used, such as interferon + 5-fluorouracil (IFN + 5-FU), low-dose 5-FU + cisplatin, and cisplatin alone. Previous studies comparing the efficacy of HAIC and sorafenib against advanced HCC have reported better tumor response, longer OS, and longer PFS in HAIC-treated patients. Even in the event of resistance to systemic chemotherapy, a local high-dose infusion of chemotherapeutic agents into the hepatic artery can be effective (16). More recently, other agents achieved better survival outcomes than sorafenib in advanced HCC, such as the FOHAIC-1 trial, Arterial Chemotherapy of Oxaliplatin Plus Fluorouracil (31). Thus, HAIC is a safe and effective alternative to sorafenib for advanced HCC patients (32–35). However, the efficacy of HAIC in HCC patients with PVTT is poorly recognized in Western countries. The latest National Comprehensive Cancer Network (NCCN) guidelines do not recommend HAIC for advanced HCC. To this end, we conducted a meta-analysis to evaluate the efficacy and safety of HAIC versus sorafenib in HCC patients with PVTT and provide an evidence-based reference for this treatment option.

According to our results, the HAIC group showed higher CR, PR, ORR, and DCR rates than the sorafenib group, corresponding to significantly lower PD rates in the former. Tumor response rates based on the Response Evaluation Criteria in Solid Tumors (RECIST) suggest that HAIC is superior to sorafenib. However, the efficacy of antineoplastic agents should be ascertained depending on the direct evidence of clinical benefits, such as improved survival, improved quality of life, or reductions in cancer-related symptoms. These survival benefits are sometimes not predicted by the indicators of tumor response. Therefore, the long-term outcomes were evaluated. Our results showed that the OS and PFS rates were also higher in the HAIC group compared to the sorafenib group, indicating that HAIC does confer long-term survival benefits to advanced HCC patients with PVTT.

Subgroup analyses were also conducted based on the regions and types of PVTT in the included studies. The main etiological factors of HCC differed between the countries and regions (7). Four studies (16, 24, 25, 27) were conducted in Korea, where the main etiological factor was hepatitis B virus infection. In contrast, hepatic C virus infection and excessive alcohol consumption were the main etiological factors of HCC in Japan and Egypt (23, 26, 28, 29). Since the pathological basis of HCC may affect the outcomes (36–38), we conducted subgroup analyses by region or etiology. We found that HAIC resulted in better ORR, DCR, OS, and DFS than sorafenib, regardless of the above factors.

In 2003, the Japan Liver Cancer Association classified PVTT into five types (Vp0 to Vp4) based on clinical features, imaging presentation, and surgical pathology (39). A previous meta-analysis (40) based on six studies showed that HAIC was more effective in patients with type Vp3-4 PVTT than in patients with type Vp2-3 PVTT (HR of OS: 0.42 vs. 0.56, HR of PFS: 0.35 vs. 0.59). Similarly, we found that HAIC provided a better survival advantage than sorafenib for patients with Vp3-4 PVTT. Since the comparison in the previous meta-analysis was not based on the tumor response, further subgroup analysis in our meta-analysis showed that the advantage of PD rates was lower in patients with HCC involving the main portal vein (Vp3-4) than in patients with thrombus in the terminal portal vein (Vp2-4). Thus, HAIC may be more suitable for PVTT patients involving the main portal vein.

In addition, Abdelmaksoud et al. (23) employed cisplatin and adriamycin as chemotherapeutic agents for HAIC. In contrast, the other studies employed cisplatin and 5-fluorouracil as HAIC chemotherapeutic agents. We also performed a subgroup analysis of HAIC with cisplatin and 5-fluorouracil. It indicated that HAIC with cisplatin and 5-fluorouracil was superior to sorafenib in ORR, DCR, OS, and DFS.

The major adverse events associated with sorafenib were hand-foot skin reactions and hypertension. Accordingly, HAIC led to fatigue, hepatic insufficiency, and leucopenia, which may be related to the side effects of the drug itself. These adverse events were mild to moderate and could be mitigated by appropriate management, including a temporary dose reduction or other symptomatic treatment. HAIC is also associated with device-related events, such as port displacement, catheter misalignment, arterial obstruction, catheter blockage, subcutaneous hematoma, or infection. Previous studies reported that the most common adverse events of HAIC were device-related (22-35%), followed by nausea and anorexia (28-33%), hematological toxicity (11-22%), gastritis (0-26%), and diarrhea (0-13%) (41). However, with technical and operational improvements, these adverse events have reduced significantly (32). A recent study reported that only 0-4% of patients experienced catheter-related adverse events following HAIC (42). In contrast, in a recent phase 3 trial conducted in the Asia-Pacific region, the major adverse events observed in sorafenib-treated patients were hand-foot skin reactions (45%), diarrhea (25.5%), and hair loss (24.8%) (15). HAIC may be a potential alternative to sorafenib if patients cannot tolerate either type of adverse event (25).

However, HAIC has its own limitations. For example, HAIC can be more effective for limited intrahepatic lesions and less effective for extra-hepatic metastases, whereas sorafenib is a systemic therapy effective against intrahepatic and extra-hepatic lesions. HAIC is yet not recommended as a treatment standard in various guidelines due to the lack of evidence. Therefore, this meta-analysis could provide some evidence to support the treatment.

## 5 Conclusions

HAIC is associated with better tumor response rates, including higher partial response, complete response, objective response, and disease control rates, and lower progressive disease compared to sorafenib in HCC patients with PVTT. Furthermore, HAIC can achieve longer overall and disease-free survival compared to sorafenib. Adverse events differed between the two groups and may have been related to the side effects of the drug itself and were relieved by appropriate management. These findings need to be further validated by high-quality RCTs in the future.

### 5.1 Limitations

1. The studies included in the meta-analysis were retrospective, leading to inevitable selection bias. 2. Despite similarities in the HAIC procedure and equipment, the protocols differed across clinics, which may also have introduced some bias. 3. Most studies were single-center and included few cases, which also affected the validity of our results.

## Author contributions

WZ and DO was responsible for drafting the manuscript, data acquisition, and interpretation of the data. WZ, DO, ZH, and XC were responsible for the design of the study and the revision of the manuscript. WZ and DO contributed equally to this work. All authors contributed to the article and approved the submitted version.
