# Supplementary material for: Hepatic arterial infusion chemotherapy versus sorafenib for advanced hepatocellular carcinoma with portal vein tumor thrombus: An updated meta-analysis and systematic review
Source: Front Oncol. 2023 Jan 27;13:1085166. doi: 10.3389/fonc.2023.1085166 (PMC9911796; doi:10.3389/fonc.2023.1085166)
Supplement: Supplementary file 1 [file DataSheet_1.pdf]

Supplementary file 1. Search strategy

PubMed:

("HAIC"[All Fields] OR (("hepatic arterial"[Title/Abstract] AND "chemotherapy"[Title/Abstract]) OR (("Hepatic"[All Fields] OR "hepatophyta"[MeSH Terms] OR "hepatophyta"[All Fields] OR "hepatics"[All Fields]) AND ("arterialization"[All Fields] OR "arterializations"[All Fields] OR "arterialize"[All Fields] OR "arterialized"[All Fields] OR "arterializing"[All Fields] OR "arterially"[All Fields] OR "arterials"[All Fields] OR "arterie"[All Fields] OR "arteries"[MeSH Terms] OR "arteries"[All Fields] OR "arterial"[All Fields] OR "arteris"[All Fields] OR "artery"[All Fields] OR "arterious"[All Fields] OR "artery s"[All Fields] OR "arterys"[All Fields]) AND ("infusate"[All Fields] OR "infusates"[All Fields] OR "infuse"[All Fields] OR "infused"[All Fields] OR "infuser"[All Fields] OR "infusers"[All Fields] OR "infuses"[All Fields] OR "infusing"[All Fields] OR "infusion"[All Fields] OR "infusions"[All Fields]) AND ("chemotherapy s"[All Fields] OR "drug therapy"[MeSH Terms] OR ("drug"[All Fields] AND "therapy"[All Fields]) OR "drug therapy"[All Fields] OR "chemotherapies"[All Fields] OR "drug therapy"[MeSH Subheading] OR "chemotherapy"[All Fields])))) AND ("carcinoma, hepatocellular"[MeSH Terms] OR ("carcinoma"[All Fields] AND "hepatocellular"[All Fields]) OR "hepatocellular carcinoma"[All Fields] OR ("hepatocellular"[All Fields] AND "carcinoma"[All Fields]) OR "carcinoma, hepatocellular"[MeSH Terms]) AND ("Sorafenib"[MeSH Terms] OR "Sorafenib"[All Fields] OR "sorafenib s"[All Fields] OR "Sorafenib"[MeSH Terms]) AND (("portal vein"[Title/Abstract] AND "thrombus"[Title/Abstract]) OR (("portal vein"[MeSH Terms] OR ("portal"[All Fields] AND "vein"[All Fields]) OR "portal vein"[All Fields]) AND ("cysts"[MeSH Terms] OR "cysts"[All Fields] OR "cyst"[All Fields] OR "neurofibroma"[MeSH Terms] OR "neurofibroma"[All Fields] OR "neurofibromas"[All Fields] OR "tumor s"[All Fields] OR "tumoral"[All Fields] OR "tumorous"[All Fields] OR "tumour"[All Fields] OR "neoplasms"[MeSH Terms] OR "neoplasms"[All Fields] OR "tumor"[All Fields] OR "tumour s"[All Fields] OR "tumoural"[All Fields] OR "tumourous"[All Fields] OR "tumours"[All Fields] OR "tumors"[All Fields]) AND ("thrombosis"[MeSH Terms] OR "thrombosis"[All Fields] OR "thrombus"[All Fields])))

Embase:

(('liver cell carcinoma'/exp OR 'carcinoma in the liver' OR 'carcinoma of the liver' OR 'carcinoma, hepatic cell' OR 'carcinoma, hepatocellular' OR 'carcinoma, liver' OR 'carcinoma, liver cell' OR 'hepatic carcinoma' OR 'hepatic cell carcinoma' OR 'hepato-carcinoma' OR 'hepato-cellular carcinoma' OR 'hepatocarcinoma' OR 'hepatocellular carcinoma' OR 'hepatocellular carcinomata' OR 'hepatocyte carcinoma' OR 'hepatocytic carcinoma' OR 'hepatoma' OR 'hepatomata' OR 'hepatomatous' OR 'liver carcinoma' OR 'liver carcinoma rupture' OR 'liver cell carcinoma' OR 'malignant hepatoma' OR 'primary liver carcinoma') OR 'hepatocellular carcinoma' AND 'portal vein tumor thrombus') AND ('Hepatic arterial infusion chemotherapy' OR 'Hepatic arterial chemotherapy') AND ((sorafenib/exp OR '4 [4 [3 [4 chloro 3 (trifluoromethyl) phenyl]

ureido] phenoxy] n methyl 2 pyridinecarboxamide' OR 'bay 43 9006' OR 'bay 43-9006' OR 'bay 439006' OR 'bay 54 9085' OR 'bay 549085' OR 'bay43 9006' OR 'bay43-9006' OR 'bay439006' OR 'bay54 9085' OR 'bay549085' OR 'fenesa' OR 'hynap-sora' OR 'nexavar' OR 'reniloxa' OR 'revamox' OR 'rexanib' OR 'sorafeb' OR 'sorafenib' OR 'sorafenib tosilate' OR 'sorafenib tosylate' OR 'soratina' OR 'weldinin') OR sorafenib)

Cochrane Library databases:

ID Search Hits

#1 (Hepatic arterial infusion chemotherapy):ti,ab,kw (Word variations have been searched) 439

#2 (sorafenib):ti,ab,kw (Word variations have been searched) 2035

#3 MeSH descriptor: [Sorafenib] explode all trees 540

#4 #2 OR #3 2035

#5 MeSH descriptor: [Sorafenib] explode all trees 540

#6 (sorafenib):ti,ab,kw (Word variations have been searched) 2035

#7 #5 OR #6 2035

#8 (portal vein):ti,ab,kw (Word variations have been searched) 2044

#9 (thrombus):ti,ab,kw (Word variations have been searched) 2815

#10 #8 AND #9 198

#11 (portal vein tumor thrombus):ti,ab,kw (Word variations have been searched) 176

#12 #10 OR #11 198

#13 #1 AND #4 AND #7 AND #12 12
