# Supplementary material for: Hepatic arterial infusion chemotherapy versus sorafenib for advanced hepatocellular carcinoma with portal vein tumor thrombus: An updated meta-analysis and systematic review
Source: Front Oncol. 2023 Jan 27;13:1085166. doi: 10.3389/fonc.2023.1085166 (PMC9911796; doi:10.3389/fonc.2023.1085166)
Supplement: Supplementary file 2 [file DataSheet_2.pdf]

Supplementary file 2 Quality evaluation of included studies

| Study | Type of Study | Selection | | | | Comparability | | Outcome | | | |
|---|---|---|---|---|---|---|---|---|---|---|---|
| | | 1 | 2 | 3 | 4 | 5 | 6 | 7 | 8 | 9 | Score |
| Abdelmaksoud 2021 | CCS | ☆ | ☆ | ☆ | ☆ | | | ☆ | | | 5 |
| Ahn 2021 | R | ☆ | ☆ | ☆ | ☆ | | | ☆ | ☆ | | 6 |
| Choi 2018 | RCT | * | * | * | * | * | * | * | * | * | * |
| Moriguchi 2017 | R | ☆ | ☆ | ☆ | ☆ | ☆ | | ☆ | ☆ | | 7 |
| Song 2015 | R | ☆ | ☆ | ☆ | ☆ | ☆ | | ☆ | ☆ | | 7 |
| Yang 2017 | CCS | ☆ | ☆ | ☆ | ☆ | ☆ | | ☆ | ☆ | ☆ | 8 |
| Nakano 2017 | P | ☆ | ☆ | ☆ | ☆ | ☆ | | ☆ | ☆ | | 7 |
| Kawaoka 2015 | R | ☆ | ☆ | ☆ | ☆ | | | ☆ | ☆ | | 6 |

Note: R- Retrospective comparative study; PSM- Propensity score matching; LG- Laparoscopic group; OG- Open group; NA- Not available; 1. Representativeness of exposed cohort; 2. Selection of non-exposed cohort; 3.Ascertainment of exposure; 4.Outcomeof interest was not present at start of study; 5. Study controls for age, sex, and marital status; 6. Study controls for any additional factors; 7.Assessment of outcomes; 8. Follow-up long enough for outcomes to occur; 9. Adequacy of follow-up; R: Retrospective comparative study; PSM: Propensity score matching.
