# Supplementary figures and images for: Hepatic arterial infusion chemotherapy versus sorafenib for advanced hepatocellular carcinoma with portal vein tumor thrombus: An updated meta-analysis and systematic review
Source: Front Oncol. 2023 Jan 27;13:1085166. doi: 10.3389/fonc.2023.1085166 (PMC9911796; doi:10.3389/fonc.2023.1085166)

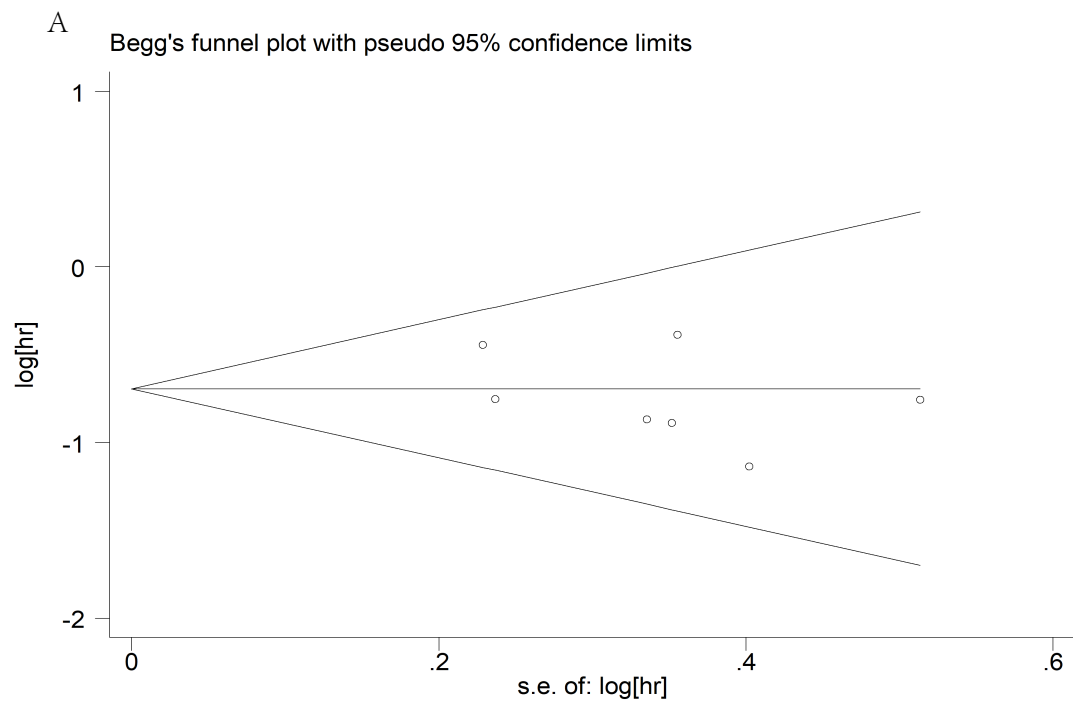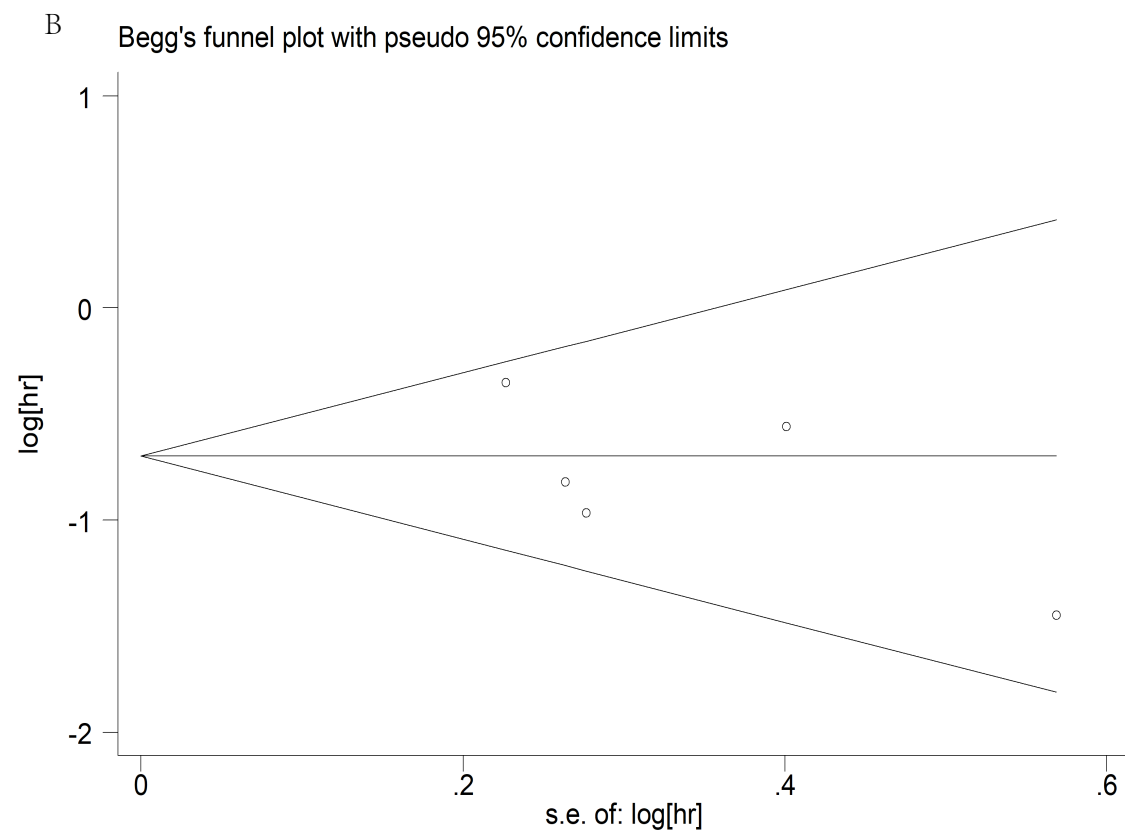

Supplementary file 3 Funnel plot of A. Overall survival, and B. Overall disease-free survival

Supplement: Supplementary file 3 [file DataSheet_3.pdf]
