# Supplementary material for: Hepatic arterial infusion chemotherapy versus sorafenib for advanced hepatocellular carcinoma with portal vein tumor thrombus: An updated meta-analysis and systematic review
Source: Front Oncol. 2023 Jan 27;13:1085166. doi: 10.3389/fonc.2023.1085166 (PMC9911796; doi:10.3389/fonc.2023.1085166)
Supplement: Supplementary file 4 [file DataSheet_4.pdf]

Supplementary file 4 Begg's and Egger's test for all included studies

Measured Outcomes	No. Studies	Begg's Test		Egger's test
		Pr>|z|☆	Pr > |z|☆☆	P>|t| ☆
**Response**				
Partial response	8	1	1	0.051
Complete response	8	0.453	0.548	0.672
Stable disease	8	0.458	0.536	0.768
Progressive disease	8	0.621	0.711	0.663
Objective response rate	8	0.805	0.902	0.052
Disease control rate	8	0.458	0.536	0.174
**Long-term outcome**				
Overall survival	7	0.293	0.368	0.305
Disease free survival	5	0.142	0.221	0.286

Note: * Values < 0.05 are considered statistically significant, which are in bold; ** Continuity corrected; NA- Not available.
